# Global Characterization of Peripheral B Cells in Parkinson’s Disease by Single-Cell RNA and BCR Sequencing

**DOI:** 10.3389/fimmu.2022.814239

**Published:** 2022-02-16

**Authors:** Pingping Wang, Meng Luo, Wenyang Zhou, Xiyun Jin, Zhaochun Xu, Shi Yan, Yiqun Li, Chang Xu, Rui Cheng, Yan Huang, Xiaoyu Lin, Lifen Yao, Huan Nie, Qinghua Jiang

**Affiliations:** ^1^ School of Life Science and Technology, Harbin Institute of Technology, Harbin, China; ^2^ Department of Neurology, First Affiliated Hospital of Harbin Medical University, Harbin, China; ^3^ Key Laboratory of Biological Big Data (Harbin Institute of Technology), Ministry of Education, Harbin, China

**Keywords:** Parkinson’s disease, B cells, scRNA-seq, scBCR-seq, adaptive immune response

## Abstract

Immune system plays important roles in the pathogenesis of Parkinson’s disease (PD). However, the role of B cells in this complex disease are still not fully understood. B cells produce antibodies but can also regulate immune responses. In order to decode the relative contribution of peripheral B cell subtypes to the etiology of PD, we performed single cell RNA and BCR sequencing for 10,466 B cells from 8 PD patients and 6 age-matched healthy controls. We observed significant increased memory B cells and significant decreased naïve B cells in PD patients compared to healthy controls. Notably, we also discovered increased IgG and IgA isotypes and more frequent class switch recombination events in PD patients. Moreover, we identified preferential V and J gene segments of B cell receptors in PD patients as the evidence of convergent selection in PD. Finally, we found a marked clonal expanded memory B cell population in PD patients, up-regulating both MHC II genes (HLA-DRB5, HLA-DQA2 and HLA-DPB1) and transcription factor activator protein 1 (AP-1), suggesting that the antigen presentation capacity of B cells was enhanced and B cells were activated in PD patients. Overall, this study conducted a comprehensive analysis of peripheral B cell characteristics of PD patients, which provided novel insights into the humoral immune response in the pathogenesis of PD.

## Introduction

Parkinson’s disease (PD) is a progressive central nervous system disorder that affects the movement (1). The main motor symptoms are rigidity, tremor, slow movement, and difficulty in walking (1). Mental and behavioral changes may also accompanied with sleep problems, depression, memory difficulties, and fatigue (1). It is estimated that 1% of people over the age of 60 suffer from PD (2, 3). About 5 to 10 percent of patients are diagnosed before the age of 50 (4, 5). Overall, about 10 million people around the world currently suffer from PD (6), and up to 80 percent of PD patients will eventually develop dementia (7).

The pathological hallmarks of PD are α-synuclein aggregation and Lewy body formation, resulting in the gradual loss of dopaminergic neurons in the substantia nigra (8). Increasing studies have shown that immune system dysfunction plays a critical role in PD pathophysiology (9). Specific variants in the HLA region are associated with PD (10, 11), and α-synuclein specific T cells were found to be involved in the pathogenesis of PD (12, 13). The levels of activated T cells are increased both in the blood and cerebrospinal fluid (CSF) of PD patients (14, 15), and T cells can also be detected in the midbrains of PD patients (16). The potential role of B cells in PD is also emerging (17). Chronic and acute MPTP administration alleviated DA neuronal loss and behavioral disorders in RAG2 knockout mice lacking both T and B cells (16, 18). IgG deposits on dopaminergic neurons has been observed in PD patients, and Lewy bodies were also coated with IgG, indicating that dopaminergic neurons might be the targets of these immunoglobulins (19). In addition, elevated levels of anti-α-synuclein antibodies can also be detected in the blood and cerebrospinal fluid of PD patients (20, 21). MPTP-treated mice produced natural and nitrated α-synuclein antibodies (22). IgG obtained from PD patients caused selective dopaminergic neuron loss (23). Although these evidences indicate that humoral immunity plays a potential role in PD, the relative contribution of peripheral B cell subtypes to the etiology of PD is still unclear.

B cells produce antibodies but can also regulate immune responses. Since infiltrating B cells have not been detected in the brains of PD patients (16), B cells may participate in central inflammation through their activities in the periphery. In this study, we conducted single-cell RNA and BCR sequencing to systematically characterize the cellular composition, immunoglobulin isotypes, preferential V and J gene segments and clonal expansion of peripheral B lymphocytes in PD patients. This large-scale single-cell expression and immune profiling data of B cells can be used as valuable resources to study the basic humoral immune response in the disease pathogenesis and potentially guide the effective diagnosis and immunotherapy strategies for PD.

## Results

### Single-Cell RNA and BCR Profiling of B Cells in Parkinson’s Disease

**Figure 1:**
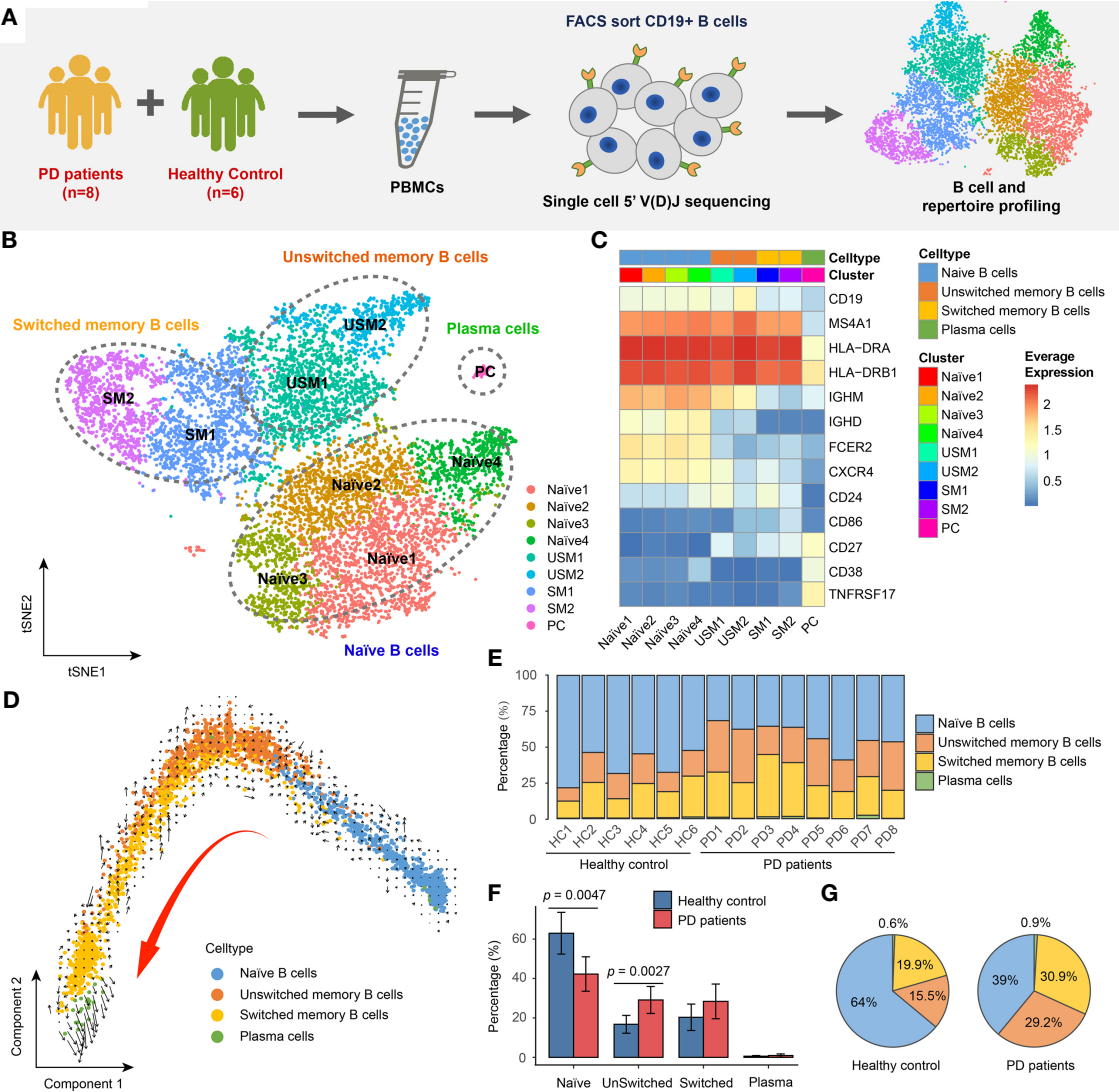
Landscape of Peripheral blood B cells in Parkinson’s disease revealed by single-cell transcriptome sequencing. **(A)** Overview of experimental design. CD19+ B cells were sorted by FACS and then subjected to single-cell RNA and V(D)J sequencing. **(B)** tSNE projection of 10,466 single B cells, showing 9 distinct clusters. **(C)** Heatmap shows the average logCPM of classical marker genes for all 9 cell clusters. **(D)** The velocities are visualized on the first two principal components calculated by monocle. **(E)** Bar chart shows the percentages of four major types of B cell in each sample. **(F)** Bar plot shows the average percentages of four major B cell types in PD patients and healthy controls. Error bars represent the standard deviation. **(G)** Pie charts show the percentage composition of B cells in PD patients and healthy controls.

We comprehensively analyzed the single-cell RNA and BCR profiling of B cells in the blood of patients with PD and healthy controls (**Figure 1A**). Detailed information of PD patients was described in **Supplementary Table S1**. Peripheral blood mononuclear cells (PBMCs) were isolated from fresh blood of 8 PD patients and 6 healthy controls. CD19+ B lymphocytes were sorted by flow cytometry, and single-cell 5’ gene-expression libraries and V(D)J enriched libraries were prepared using a 10x Genomics single-cell immune profiling workflow. After removing low-quality cells, we finally obtained single-cell expression data for 10,466 B cells, comprising 6,681 cells (mean: 835 cells) for PD patients and 3,785 cells (mean: 631 cells) for healthy controls (**Supplementary Table S1**). In addition, we obtained 13,957 single-cell paired BCRs, comprising 8,704 BCRs (mean: 1088 BCRs) from PD patients and 5,253 BCRs (mean: 876 BCRs) from healthy controls (**Supplementary Table S1**). 10,206 B cells have paired gene expression and BCR profiles (**Supplementary Table S1**).

### Altered B Cell Composition and Transcriptome in Parkinson’s Disease

In order to reveal the internal cellular composition and functional status of peripheral blood B cells, different B cell populations were identified using an unsupervised clustering approach embedded in Seurat (24, 25). B cells were visualized in t-distributed stochastic neighbor embedding (t-SNE) based on the gene expression profiling. In total, we identified 9 distinct clusters representing different B cell subtypes (**Figure 1B**). B cells were annotated by manually checking the cell-identity marker expression and their global similarity with the gene expression of reference datasets (26–30) by SingleR (31). Spearman correlation between single cell and bulk RNA-seq expression profiles were computed, and the cell labels were transferred from bulk RNA-seq datasets to every single cell. Each dataset was used separately to calculate the correlation. In most cases, a cluster was annotated by the labels of majority of cells from that cluster (**Supplementary Figure S1**). We identified the major cell types of B cells, including: naïve B cells (IgD+CD27-) (Naïve1, Naïve2, Naïve3, Naïve4 clusters), unswitched memory B cells (IgD+CD27+) (USM1, USM2 clusters), switched memory B cells (IgD-CD27+) (SM1, SM2 clusters) and plasmablast/plasma cells (IgD-CD27hi) (PC cluster) (**Figures 1B, C**). For unswitched memory B cells, compared to USM1 subset, USM2 overexpressed several marker genes related to B cell activation, proliferation, and differentiation, such as FCRL5 (32), EGR1 (33) and CD86 (34) (**Supplementary Figure S2A**). For switched memory B cells, SM1 and SM2 had distinct gene expression patterns (**Supplementary Figure S2B**). SM1 overexpressed CD24, which induces apoptosis in human B cells through interactions with glycolipid-enriched membrane domains (35), while SM2 highly expressed CD99, an activation-associated molecule that is upregulated in recently activated lymphocytes (36) (**Supplementary Figure S2B**). Overall, the USM2 and SM2 subsets of memory cells appear to be activated B cell populations, and we will focus on their composition and functional status in patients with Parkinson’s disease. To further understand the relationships among B cell subsets, we used Monocle (37) to perform pseudo-time ordering for these cells and visualize them on the first two principal components (**Figure 1D**). A process of transformation from naïve B cells to unswitched and switched memory B cells followed by plasma cells is clearly observed (**Figure 1D**). And this lineage directionality was further confirmed by RNA velocities (**Figure 1D**).

In order to understand the composition of B cell populations in PD patients, we compared the percentage of B cell subsets in PD patients and healthy controls. B cells exhibit a specific composition in PD patients (**Figure 1E**). We observed significant increase of unswitched memory B cells (Wilcox test, two-sided, *p* = 0.0027) and significant decrease of naïve B cells (Wilcox test, two-sided, *p* = 0.0047) in PD patients compared to healthy controls (**Figure 1F**). In PD patients, naïve B cells accounted for 39% of the total B cells, while in healthy controls, this proportion rose to 64% (**Figure 1G**). While, unswitched memory and switched memory B cells accounted for 60.1% of total B cells in PD patients, which was nearly twice of that in healthy controls (**Figure 1G**).

### Immunoglobulin Isotypes and Class-Switching Events of BCRs in PD Patients

**Figure 2:**
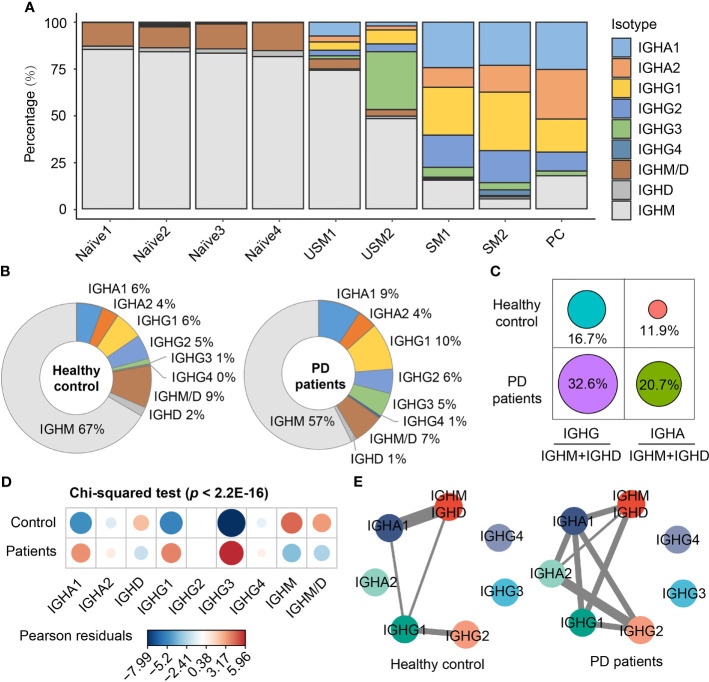
Immunoglobulin isotypes and class-switching events of BCRs in PD patients and healthy controls. **(A)** Immunoglobulin isotype distribution in each B cell subsets. **(B)** Pie chart shows the percentage composition of Ig isotypes in PD patients and healthy controls. **(C)** The ratio of IgG to IgM/D and IgA to IgM/D between PD patients and healthy controls. **(D)** Bubble chart shows the Pearson’s residuals of sample groups from Ig isotypes. Red circles indicate an over-representation, and blue circles indicate an under-representation. **(E)** Class-switching events in PD patients and healthy controls. The thickness of the line indicates the number of sharing clonotypes between two Ig isotypes.

To explore B cell immunoglobulin (Ig) repertoire, we investigated Ig heavy-chain isotypes for PD patients and healthy controls. Four main types of Ig were detected from scBCR-seq data, including IgD, IgM, IgA and IgG. IgM had the largest proportion in naïve B cells and unswitched memory B cells, while IgG and IgA were mainly distributed in switched memory B cells and plasma cells (**Figure 2A**). In PD patients, IgM accounted for 57% of the total BCR isotypes, while in healthy controls, this proportion rose to 67% (**Figure 2B**). In addition, IgG accounted for 22% of total BCR isotypes in PD patients, which was 1.8 folds of that in healthy controls (**Figure 2B**). The ratios of IgG to IgM/D and IgA to IgM/D in PD patients were almost twice of those in healthy controls (**Figure 2C**). Chi-squared test further confirmed the significant association between disease and different BCR isotypes (Pearson’s chi-squared test, X-squared = 244.06, df = 8, simulated p < 2.2E-16, **Figure 2D**). The isotypes IgG1 and IgG3 were significantly associated with PD patients, while IgM and IgD was significantly associated with healthy controls (**Figure 2D**). Since antibodies that undergo Ig class switch recombination (CSR) and somatic hypermutation (SHM) have higher affinity and longer-lasting protection (38), we assigned BCRs to clonotypes and performed comparison analysis of CSR events in each clonotype between PD patients and healthy controls. Clonotypes were defined by clustering Ig sequences using the DefineClones function in Change-O toolkit (39). We observed more CSR events in PD patients, especially IgM to IgG and IgA to IgG (**Figure 2E**). These changes suggest an activated humoral immune response in the peripheral blood of PD patients.

### Preferential V and J Gene Segments of BCRs in PD Patients

In order to search for the evidence of convergent antibody evolution in PD, we compared the preferential gene segment usage in V(D)J rearrangements between PD patients and healthy controls. B cells undergo V(D)J recombination of variable (V) and joining (J) gene segments in the light (L) chain (κ and λ), and of variable (V), diversity (D), and joining (J) gene segments in the heavy (H) chain in order to generate diverse repertoires of B cell receptors capable of recognizing a wide range of pathogen epitopes (40).

**Figure 3:**
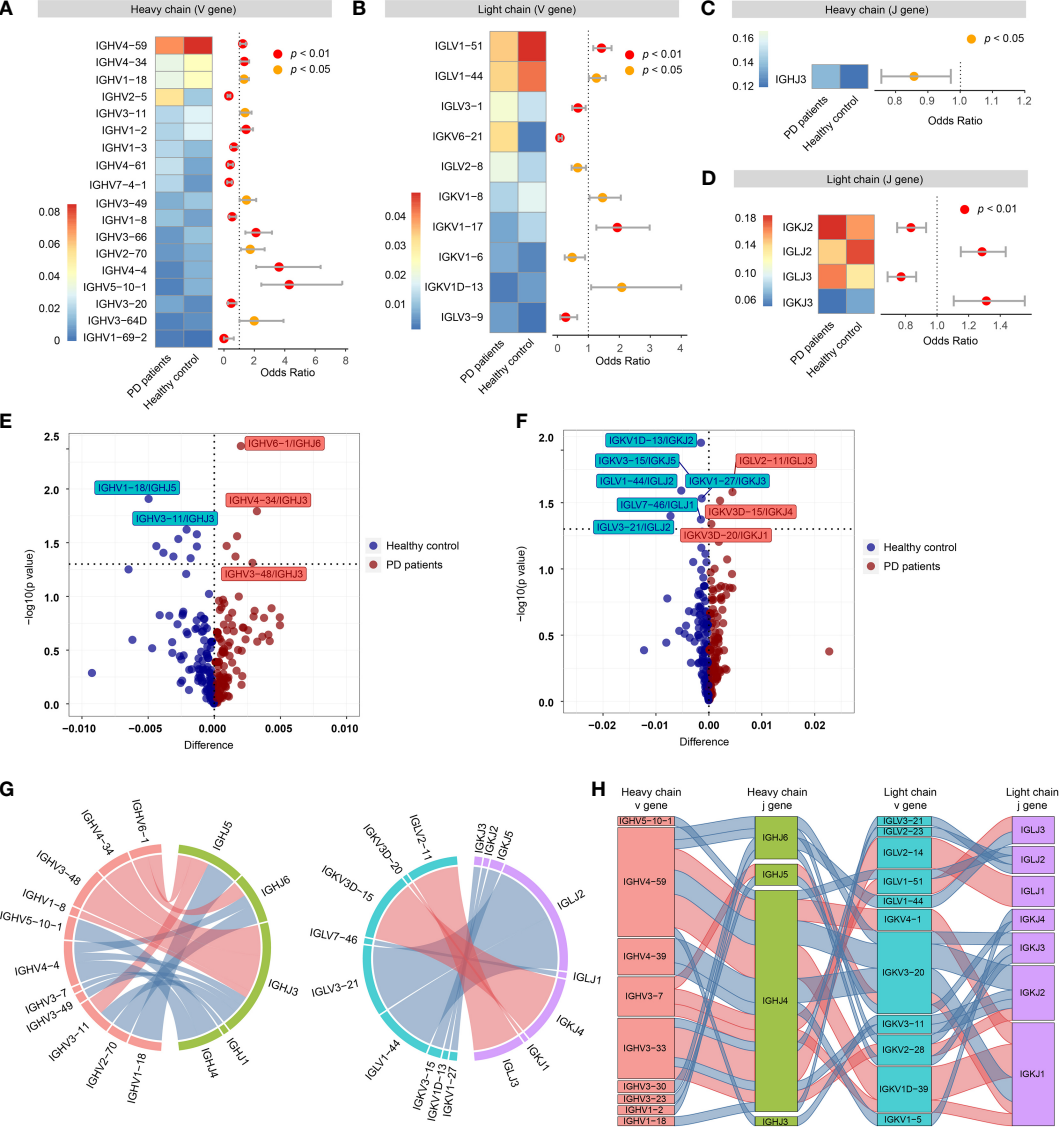
Preferential variable (V) and joining (J) gene segments in the heavy and light chain of BCRs in PD patients. **(A)** Differential usage (Fisher’s exact test) of V_H_ gene segments between PD patients and healthy controls. **(B)** Similar to A, differential usage of V_L_ gene segments. **(C)** Similar to A, differential usage of J_H_ gene segments. **(D)** Similar to A, differential usage of J_L_ gene segments. **(E)** Volcano plot shows the significant different frequency of heavy chain VJ pairs of PD patients compared to healthy controls. **(F)** Similar to E, volcano plot shows the significant different frequency of light chain VJ pairs. **(G)** Circos plots show the differential heavy (left) and light (right) VJ pairs in PD patients and healthy controls. Red links represent PD specific VJ pairs, and blue links represent healthy control specific VJ pairs. **(H)** Sankey diagram shows significant different frequency of heavy-light VJ pairs in PD patients and healthy controls. Red links represent PD specific pairs, and blue links represent healthy control specific pairs.

For V_H_ gene segments, the frequencies of 18 V_H_ gene segments were significantly different between PD patients and healthy controls, of which, 7 V_H_ gene segments (IGHV2-5, IGHV1-3, IGHV4-61, etc.) increased in PD patients, while 11 V_H_ gene segments (IGHV4-59, IGHV3-34, IGHV1-18, etc.) increased in healthy controls (**Figure 3A**). For V_L_ gene segments, 10 V_L_ gene segments were significantly different between PD patients and healthy controls, of which 5 V_L_ gene segments (IGLV3-1, IGKV6-21, IGLV2-8, etc.) increased in PD patients and 5 V_L_ gene segments (IGLV1-51, IGLV1-44, etc.) increased in healthy controls (**Figure 3B**). For J_H_ gene segments, only IGHJ3 was significantly increased in PD patients (**Figure 3C**). For J_L_ gene segments, IGKJ2 and IGLJ3 were significantly increased in PD patients, while IGLJ2 and IGKJ3 were significantly increased in healthy controls (**Figure 3D**).

Then, we further compared the VJ pairing of heavy and light chains separately. For heavy chain, 6 VJ_H_ pairs (IGHV6-1/IGHJ6, IGHV4-34/IGHJ3, etc.) were significantly increased in PD patients, while 9 VJ_H_ pairs (IGHV1-18/IGHJ5, IGHV3-11/IGHJ3, etc.) were significantly increased in healthy controls (**Figures 3E, G**). For light chain, 3 VJ_L_ pairs (IGLV2-11/IGLJ3, IGKV3D-15/IGKJ4 and IGKV3D-20/IGKJ1) were significantly increased in PD patients, while 6 VJ_L_ pairs (IGKV1D-13/IGKJ2, IGKV3-15/IGKJ5, etc.) were significantly increased in healthy controls (**Figures 3F, G**). Of all the heavy-light VJ pairs, 20 heavy-light VJ pairs were significantly different between PD patients and healthy controls, of which 7 heavy-light VJ pairs increased in PD patients, others decreased (**Figure 3H**).

### A Marked Clonal Expansion of Memory B Cells in PD Patients

**Figure 4:**
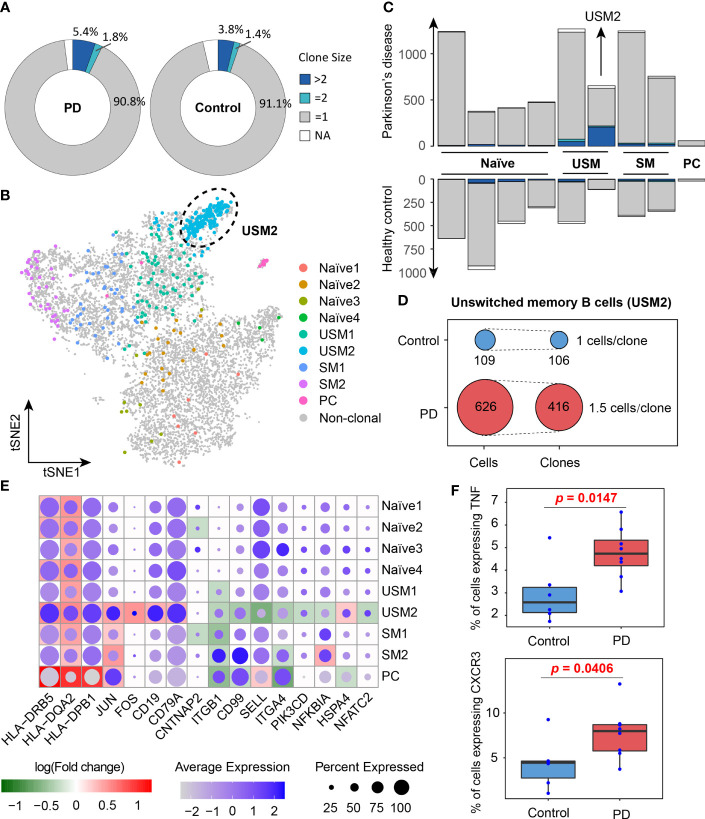
Clone expansion of B cells in Parkinson’s disease. **(A)** Pie charts show the distribution of clonotypes grouped by clone size (NA, = 1, = 2, >2, NA represents cells with no BCR sequence detected). **(B)** tSNE plot shows the distribution of clonally expanded B cells. **(C)** Bar plots show the distribution of clonotypes grouped by clone size in PD patients and healthy controls. **(D)** Clonotype diversity of unswitched memory B cells (USM2) in PD patients and healthy controls. **(E)** A global view of the differentially expressed genes related to B cell receptor signaling pathway in each cluster. The size of the dot represents the percentage of cells expressing the gene in each cluster, while the color represents the average gene expression value. Background heatmap shows the log-transformed fold-change of DEGs between PD patients and healthy controls. **(F)** Box plots show the percentages of B cells expressing TNF and CXCR3 in PD patients and healthy controls. Each dot represents a sample.

In order to better understand the activated B cell types in PD, we conducted a comparison analysis of the B cell clonal expansion between PD patients and healthy controls. In total, we detected 13,957 BCRs, forming 12,938 unique clonotypes, of which 357 clonotypes detected in at least two cells, indicating clonal expansion of peripheral blood B cells. 7.2% (5.4% + 1.8%) of B cells were clonally expanded in PD patients, which was higher than 5.2% (3.8% + 1.4%) in healthy controls (**Figure 4A**). The UMAP plot shows that clonal expansion mainly occurs in memory B cells, especially the unswitched memory B cell cluster USM2 (**Figure 4B**). Fisher’s exact test further confirmed the significance of the clonal expansion of unswitched memory B cell cluster USM2 (Fisher’s exact test, two-sided, FDR = 1.29E-12) (**Figures 4B, C**). In PD patients, B cell cluster USM2 tend to have much larger clonotypes with 416 clonotypes detected from 626 B cells (an average of 1.5 B cells per clonotype), while in healthy controls the average clone size was 1 (106 clonotypes out of 109 B cells) (**Figure 4D**). Antibodies produced by unswitched and switched memory B cells from the same clonotypes retain affinity for the same antigens (41). Since antibody class switching diversifies the effector properties of antibodies (41), clonal expansion of memory B cells (especially unswitched memory B cells) in PD patients may be a strategy to cope with the increasing central nervous system inflammation.

To investigate the function of B cell populations in PD, we conducted differentially expressed analysis for each B cell cluster between PD patients and healthy controls (**Supplementary Table S2**). Gene Ontology and KEGG pathway enrichment analyses of the differentially expressed genes (DEGs) were performed (**Supplementary Table S2**). DEGs enriched in B cell receptor signaling pathway (hsa04662), antigen processing and presentation (hsa04612) and cell adhesion molecules (hsa04514) were selected to further view their expression profiles in each cluster (**Figure 4E**). We observed that MHC II genes (HLA-DRB5, HLA-DQA2 and HLA-DPB1) were significantly overexpressed in B cells of PD patients, especially in memory B cells and plasma cells, indicating their antigen presentation function were enhanced in PD patients (**Figure 4E**). It is generally believed that B cells as APCs are less effective than dendritic cells and other myeloid cells, but when the antigen recognized by BCR is presented to T cells that recognize the same antigen, the antigen presentation efficiency of memory B cells increases (42–44). In addition, memory B cells and plasma cells in PD patients also up-regulated activator protein 1 (AP-1) transcription factors (JUN and FOS) (**Figure 4E**), which controls a number of cellular processes including differentiation, proliferation, and apoptosis during B-cell activation (45). These results suggest that the antigen presentation capacity of B cells was enhanced and B cells were activated in PD patients.

## Discussion

Recently, neuroinflammation has attracted increasing attention due to its potential in the development of novel molecular biomarkers and targeted therapies (46, 47). In has been shown that both innate and acquired immunity play important roles in neuroinflammation in the progression of PD (48, 49). Previous studies have shown that acquired immunity, especially T cell immunity, plays a key role in the immune system dysfunction of PD (50–52), but the function of B cells in PD is still not fully understood (17).

In this study, we comprehensively characterized the B cell population in patients with PD. Although some studies have reported that the level of total B cells remains unchanged or even decreased in patients with PD (53–55), our study shows that the B cell subpopulation structure has changed significantly. We observed significantly decreased naïve B cells and significantly increased unswitched memory B cells (especially USM2 subset) in PD patients compared to healthy controls. Notably, the two unswitched memory B cell clusters, USM1 and USM2, both harbored IGHD and highly expressed the memory marker CD27, but had distinct gene expression patterns (**Figure S2A**). USM2 overexpressed several B cell activation-related genes, such as FCRL5, EGR1 and CD86. FCRL5+ memory B cells are optimally responsive cells (32), have more extensive proliferative history (56), and are committed to becoming plasmablasts (57). EGR1 participates in B cell maturation as a positive regulator (33). CD86 is usually upregulated after B cell activation, which can then be further activated and in turn activate T cells (34). It is reported that unswitched memory B cells show faster and stronger re-stimulation potential and are involved in early inflammatory response (58). In this study, the USM2 subset has strong activation features and high proliferative potential. We speculate that it may be an important participant in the humoral immune response of Parkinson’s disease, and may eventually contribute to the production of infiltrating antibodies in the brain of PD patients. Their potential role in Parkinson’s disease deserves further investigation.

We also discovered increased IgG and IgA isotypes and more frequent CSR events in PD patients compared to healthy controls. B cells have not been detected in the brains of PD patients (16), but IgG deposition had been observed around dopaminergic neurons and Lewy bodies (19). Increased circulating IgG and IgA isotypes may contribute to the IgG detected in the brain of PD patients. Moreover, the preferential V and J gene fragments of B cell receptors in PD patients found in this study also further provide the evidence of convergent selection in PD. HLA-DRB5 (59, 60), HLA-DQA2 (60, 61) and HLA-DPB1 (62) have been reported to be associated with PD, which were all up-regulated in memory B cells of PD patients compared to healthy controls in this study, suggesting the enhanced capacity of antigen presentation in B cells of PD patients.

We observed BCR-induced activation of the AP-1 transcription factor up-regulated in PD patients, indicating B cell activated in PD patients. B cells produce antibodies but can also regulate immune responses. We noted that PD patients had an increased proportion of TNFα-producing B cells compared with healthy controls (Wilcox test, one-sided, *p* = 0.0147, **Figure 4F**). High levels of soluble TNFα have been detected in the cerebrospinal fluid and postmortem brains of PD patients as well as in animal models of PD (63–66). And the expression level of TNFα in cerebrospinal fluid is a candidate risk biomarker for the detection of PD at the prodromal stage (67). Anti-TNFα therapy protects dopaminergic neurons (68) and reduces the incidence of Parkinson’s disease (69). These findings suggest that TNFα may be a mediator of neuronal injury and a feasible target for the treatment of PD. The relationship between the increased TNFα production in B cells and the progression of Parkinson’s disease needs further investigation. In addition, we also found that the proportion of CXCR3-expressing B cells was significantly increased in PD patients (Wilcox test, one-sided, *p* = 0.0406, **Figure 4F**), suggesting enhanced chemotaxis of B cells in PD patients. These B cell abnormalities may contribute to the development and progression of Parkinson’s disease by increasing antibody and cytokine infiltration and enhancing neuroinflammation in the central nervous system (70). Overall, our study provides a comprehensive characterization of peripheral B cells in PD patients, which provide novel insights on the humoral immune response in the pathogenesis of PD.

Although our results suggest that B cells may play a role in PD, it is not clear whether the observed changes in acquired immunity are causal or secondary to central nervous system disorder associated with the pathogenesis of PD. Further studies, such as whether anti-α-synuclein monoclonal antibody therapy is beneficial to patients with PD (71), are still needed to investigate the B cell immunity in the pathogenesis of PD.

## Materials and Methods

### Human Participants

In this study, we recruited eight PD patients (PD1-PD8, 50-70 years) and six healthy controls (HC1-HC6, 51-72 years). All participants had no obvious somatic disorders, such as cancer, autoimmune diseases, as well as mental and cognitive disorders. All participants were recruited from the First Affiliated Hospital of Harbin Medical University and obtained informed consent. This study was approved by the Ethics Committee in the First Affiliated Hospital of Harbin Medical University, and the approval number is No. 201985.

### Blood Sample Collection and Single-Cell 5’ and V(D)J Sequencing

Fresh blood samples were collected from 8 PD patients and 6 age-matched healthy controls. Then, peripheral blood mononuclear cells (PBMCs) were isolated by Percoll density gradient centrifugation. Next, CD19+ B cells were sorted using fluorescence-activated cell sorting (FACS). Single-cell 5’ gene expression libraries and V(D)J enriched libraries were prepared according to the standard protocols provided by the 10x Genomics Chromium Single Cell Immune Profiling Solution. Finally, single-cell 5’ gene expression and V(D)J libraries were sequenced on Illumina Noveseq 6000, providing 150 bp paired-end reads.

### Preprocessing of Single-Cell 5’ V(D)J Sequencing Data

Single cell 5’ gene expression and V(D)J data was processed by Cell Ranger pipeline (version 3.1.0) for each sample. Reference data files refdata-cellranger-GRCh38-3.0.0 and refdata-cellranger-vdj-GRCh38-alts-ensembl-3.1.0 downloaded from 10x Genomics official website were used in Cell Ranger analysis pipelines for single-cell transcriptome and V(D)J data processing, separately.

### Cell Quality Control

We used emptyDrops function in the R package dropletUtils (72, 73) to detect and remove empty droplets. Doublets were detected by R package DoubletFinder (74) with default parameters. After removing empty droplets and doublets, low-quality cells were identified based on the median absolute deviation (MAD) using isOutlier function in the R package scater (75). Three matrics were used to detect low-quality cells: 1) Total UMI counts per cell (library size); 2) Total detected genes per cell; 3) The proportion of mitochondrial gene counts. Please see Zhang et al. (76) for details. Finally, genes with more than 1 transcript in at least two cells were retained for further analysis.

### Dataset Integration and Unsupervised Clustering

Batch effects were removed, and datasets from each sample were integrated using the standard Seurat v3 integration workflow. First, raw counts of each sample were normalized using a global-scaling normalization method NormalizeData in R package Seurat (24, 25). This method normalizes the gene expression values for each cell by the total UMI counts in the sample, then multiplies this value by a scale factor (10,000 by default), and log-transforms the result. Then FindVariableFeatures function in Seurat (24, 25) was used to identify highly variable genes to further reduce the dimensionality of the data. Next, ‘anchors’ between pairs of samples were identified and used to harmonize the datasets. Finally, cell cycle effects were calculated by CellCycleScoring function and regressed by ScaleData function in Seurat (24, 25).

### Cell Type Annotation

Gene expression markers were identified by FindAllMarkers function in Seurat, which performs Wilcoxon Rank Sum test to identify differentially expressed genes for each cluster. Then, SingleR (31) was applied to enhance cell type annotation by calculating global similarity of gene expression between each cell and the reference datasets. Five bulk RNA-seq datasets of purified immune cells [The Database for Immune Cell Expression (26), Monaco Immune Cell Data (27), Human Primary Cell Atlas (28), BLUEPRINT database (29) and Novershtern Hematopoietic Data (30)] were selected as reference datasets for expression similarity based cell annotation. Spearman correlation between single cell and bulk RNA-seq expression profiles were computed, and the cell labels were transferred from bulk RNA-seq datasets to every single cell. Each dataset was used separately to calculate the correlation. Cells types were finally annotated by manually checking the cell-identity marker expression and their global similarity with the gene expression of reference datasets.

### Pseudo-Time Reconstruction

Firstly, single-cell trajectory analysis was conducted by Monocle 2 (version 2.14.0), which reconstructs trajectories based on tracking expression changes. Given that the direction of pseudo-time is arbitrary, we selected naïve B cells as the beginning of the trajectory. A process of transformation from naïve B cells to unswitched and switched memory B cells followed by plasma cells is clearly observed (**Figure 1D**). Then, RNA velocity was calculated to further characterize cell fate decisions and lineage relationships. RNA velocity was estimated by velocyto pipelines (77). Finally, the velocities were visualized on the first two principal components calculated by monocle.

### Differential Gene Expression Analysis

Differentially expressed genes (DEGs) was identified by FindMarkers function in Seurat (24, 25) using default parameters. Only genes that were detected in at least 10% of the cells in one of the two groups were tested. When calculating the logFC value, the average expression value of each group was added by 1 (where 1 represents a pseudocount) and then divided, followed by logarithmic conversion. *P* values were estimated using two-sided Wilcoxon test, and FDR was corrected using BH. DEGs between PD patients and healthy controls as well as gene expression markers of each cell subtypes were combined to evaluate the function of B cell subtypes in the humoral immune response of PD.

### Statistical Analysis

All statistical analyses and visualization were performed using R the statistical programming language (version 4.0.3). Two sample tests were performed using two-sided Wilcoxon rank sum test. If multiple tests were performed for a single analysis, we used BH procedure to correct for FDR.

## Data Availability Statement

Single-cell RNA and BCR sequencing data are available at GEO (Gene Expression Omnibus) under accession number GSE194245. Custom scripts used to analyze data in this article are available upon request to the Lead Contact.

## Ethics Statement

The studies involving human participants were reviewed and approved by the Ethics Committee in the First Affiliated Hospital of Harbin Medical University (Approval number: No. 201985). The patients/participants provided their written informed consent to participate in this study.

## Author Contributions

QJ, LY, and HN conceived the project. SY collected the blood samples. PW contributed to data analysis. ML, WZ, XJ, ZX, YL, CX, RC, YH, and XL provided the technical support. PW wrote the manuscript. QJ reviewed and edited the manuscript. All authors contributed to manuscript revision, read, and approved the submitted version.

## Funding

This work was funded by the National Natural Science Foundation of China (Nos. 62032007 to QJ, and Nos. 62072143 to LY).

## Conflict of Interest

The authors declare that the research was conducted in the absence of any commercial or financial relationships that could be construed as a potential conflict of interest.

## Publisher’s Note

All claims expressed in this article are solely those of the authors and do not necessarily represent those of their affiliated organizations, or those of the publisher, the editors and the reviewers. Any product that may be evaluated in this article, or claim that may be made by its manufacturer, is not guaranteed or endorsed by the publisher.

## Supplementary Material

Supplementary Figure 1 Heatmap showing the percentage of cells in each cluster annotated by three purified bulk RNA-seq datasets.

Supplementary Figure 2 Volcano plot showing differentially expressed genes (DEGs) between unswitched memory B cells USM1 and USM2 **(A)**, as well as between switched memory B cells SM1 and SM2 **(B)**. The x-axis represents the fold change between groups, and the y-axis represents *P* values.

Supplementary Table 1 Summary of single-cell RNA and BCR sequencing data.

Supplementary Table 2 Differentially expressed genes and functionally enriched GO terms and KEGG pathways between PD patients and healthy controls in each B cell cluster.
