# Supplementary figures and images for: Global Characterization of Peripheral B Cells in Parkinson’s Disease by Single-Cell RNA and BCR Sequencing
Source: Front Immunol. 2022 Feb 16;13:814239. doi: 10.3389/fimmu.2022.814239 (PMC8888848; doi:10.3389/fimmu.2022.814239)

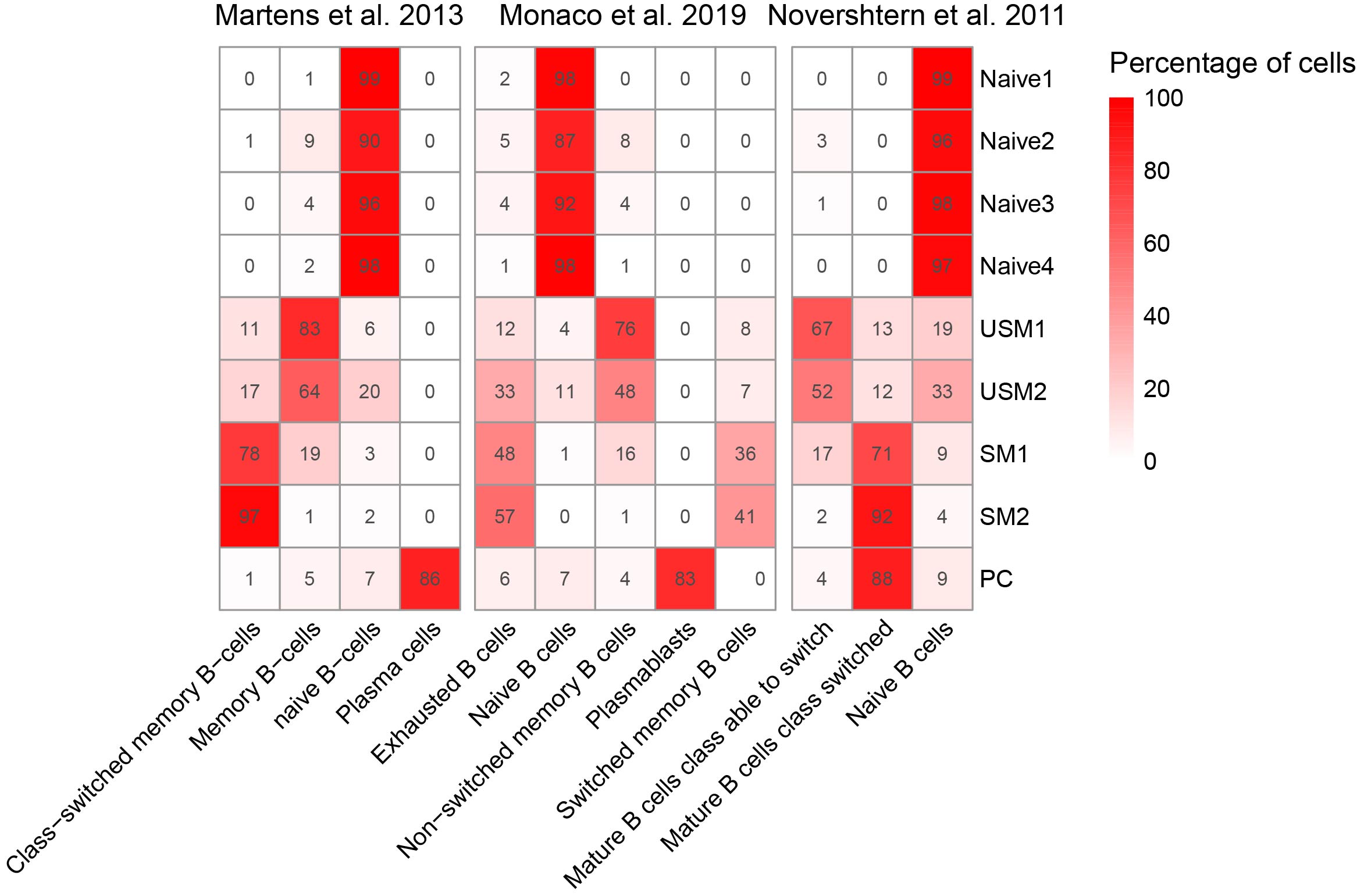

Supplement: Supplementary Figure 1 — Heatmap showing the percentage of cells in each cluster annotated by three purified bulk RNA-seq datasets. [file Image_1.jpeg]

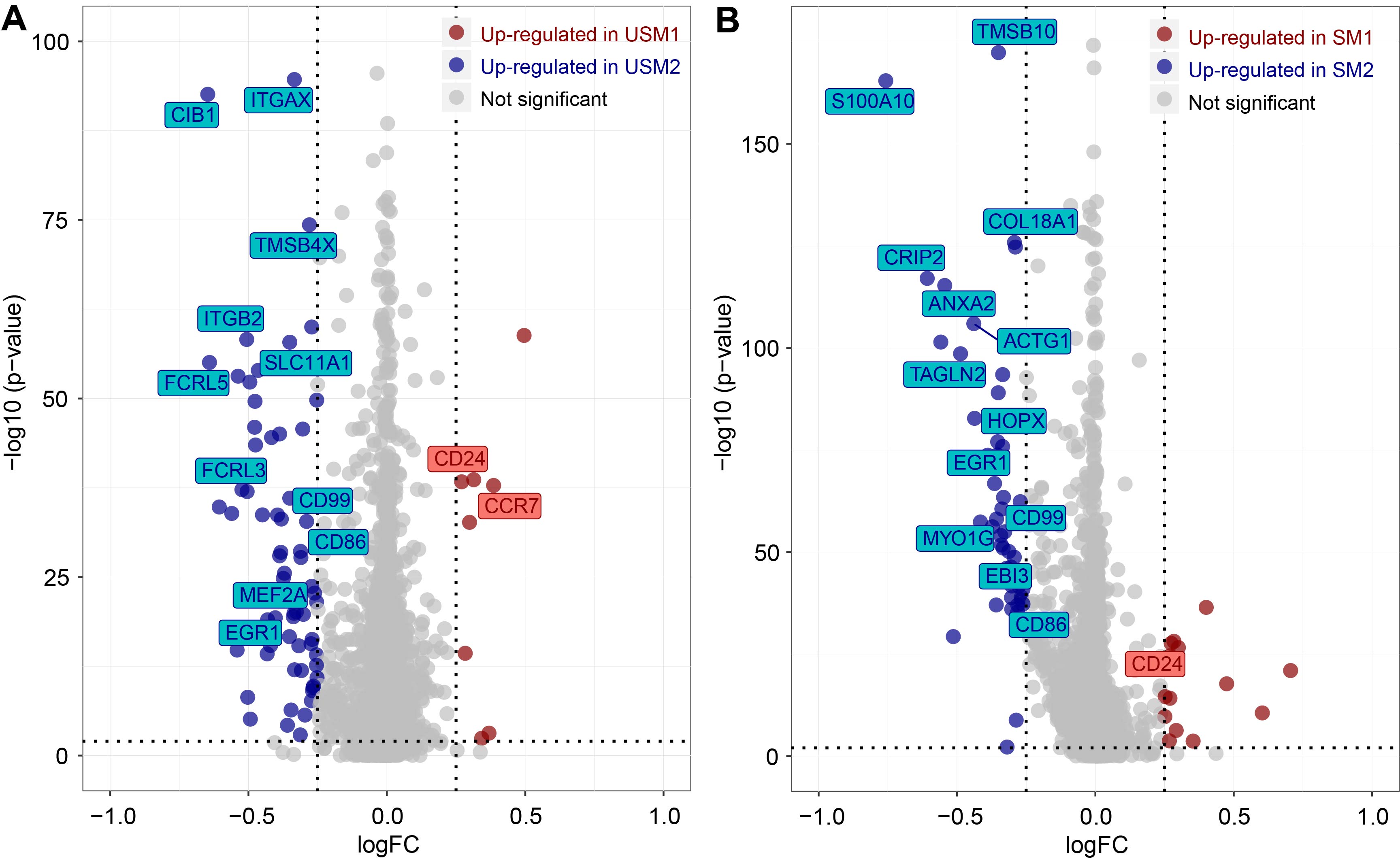

Supplement: Supplementary Figure 2 — Volcano plot showing differentially expressed genes (DEGs) between unswitched memory B cells USM1 and USM2 (A), as well as between switched memory B cells SM1 and SM2 (B). The x-axis represents the fold change between groups, and the y-axis represents P values. [file Image_2.jpeg]
